# Demographic aspects of first names

**DOI:** 10.1038/sdata.2018.25

**Published:** 2018-03-06

**Authors:** Konstantinos Tzioumis

**Affiliations:** 1 Office of the Comptroller of the Currency (OCC), 400 7th Street SW, Washington, D.C. 20219, USA

**Keywords:** Economics, Interdisciplinary studies

## Abstract

We introduce a list that offers information on the relation between first names and race or ethnicity. Drawing information from mortgage applications, the list includes 4,250 first names and information on their respective count and proportions across six mutually exclusive racial and Hispanic origin groups. These six categories are consistent with the categories used in the Census Bureau's list on surnames' demographic information. Also, just like the Census Bureau's list of surnames, the list of first names is highly aggregated, so as to not identify any specific individuals.

## Background & Summary

Classifying individuals into racial or ethnic groups has been an essential part of academic and policy studies in a wide range of areas, such as demography, medicine, public health policy, and discrimination. A common denominator for the great majority of these studies is the absence of any direct information on the individual's race or ethnicity, and the need to impute this information from other readily available parameters. Two avenues have evolved to address this information scarcity, namely the use of associated information to infer race or ethnicity, and the development of methodologies that combine different strands of information in an effort to improve the assignment accuracy. In terms of associated information to infer race and ethnicity, researchers typically use a person’s surname and/or geographic location based on aggregates provided by national statistical authorities, such as the Census Bureau in the United States. Also, when combining information, researchers have opted for either simple probabilistic models or a Bayesian approach, such as the Bayesian Improved Surname Geocoding (BISG)^1,2^.

This paper contributes to the first avenue by producing a comprehensive list of first names and outlining the benefits of using the first name as an additional parameter in race/ethnicity classification. This contribution is novel for a number of reasons. First, the Census offers race/ethnicity information for surnames but not for first names^3,4^. Specifically, the 2010 (2000) Census offers information only when the surname frequency exceeds 100 observations, thus containing 162,253 (151,671) surnames and covering about 90 percent of the U.S. population whose surname was recorded^5,6^. In contrast, first names have a far higher concentration rate since the top 1,000 first names, for males and females respectively, were capturing about 86 percent of the U.S. population in 1990 ref. 7. Second, using only surnames that may actually belong to the spouse decreases the accuracy of the race/ethnicity imputation for females. This point is important given the sharp increase in interracial marriages after the 1970s ref. 8. Third, we use Loan Application Registers (LARs), required by the Home Mortgage Disclosure Act (HMDA), as a data source for race/ethnicity information. This alleviates any concerns about the validity of the race/ethnicity information, since self-report is well accepted in defining an individual's racial and ethnic identity^9,10^. Fourth, the academic literature that has used first names to test outcome variations across races focuses on extreme cases that can be uniquely assigned to a specific race or ethnicity^11^. By having the full range of first names, one could test the presence of nonlinearities in the associations between racial/ethnic groups and various outcomes, as well as variations across geographies.

## Methods

### Data sources

We extract information from three distinct proprietary mortgage datasets in order to create a single unique list of first names and their corresponding proportions for race and ethnicity. The first dataset consists of mortgage applications from a lender in 2010. The second dataset is a merged dataset between HMDA and DataQuick for 2010 that excludes any loans from the lender in the first dataset (see ‘Additional information on the data merging methodology’ sub-section about the merging process and how the choice of year acts as an added quality control test for that process). The third dataset consists of mortgage applications from a subprime lender in 2007.

We combine the three datasets given that we need a substantial volume of data to reliably calculate observed probabilities (proportions), cover a large number of names, and address any geographic or customer selection aspects of a specific lender. Also, we chose the datasets in such a way that avoids double-counting since there is no overlap between datasets from an institutional perspective. From an applicant perspective, there could be an overlap if the applicant from one dataset applied for another mortgage (e.g., for refinance) in the other dataset. However, given the nature and the timing of the three datasets, we expect this overlap to be trivial. Finally, it is important to note the consistency in collecting and reporting information on race and ethnicity across the three datasets since the respective lenders comply with HMDA requirements.

### Categories for race and ethnicity

Based on the HMDA information, we initially classify applicants' race and ethnicity into twelve mutually exclusive categories. We then aggregate these categories into six categories that are based on the pre-1997 Office of Management and Budget (OMB) definitions and are reflected in the 2000 and 2010 Census surname datafiles on race and ethnicity^3,4^. This categorization unifies all Hispanics into one group regardless of race, while the remaining Non-Hispanic (henceforth ‘*NH*’) are grouped into five race categories. Overall, the six categories are ‘Hispanic or Latino,’ ‘NH White,’ ‘NH Black or African American,’ ‘NH Asian or Native Hawaiian or Other Pacific Islander,’ ‘NH American Indian or Alaska Native,’ and ‘NH Multi-race.’

Table 1 illustrates how the twelve HMDA-based categories correspond to the six OMB-based categories for race and ethnicity. Notably, there is little information loss from reducing the number of categories from twelve to six since the ‘NH Asian’ category contributes about 96 percent to the consolidated ‘NH Asian or Native Hawaiian or Other Pacific Islander’ category and the ‘Hispanic White’ category contributes about 95 percent to the consolidated ‘Hispanic’ category.

### Editing steps

The editing steps are identical for each of the three datasets mentioned earlier. Below we outline each step:

- Drop all information from the dataset except for the race and ethnicity variables, and the full name of the applicant(s). Prior to this, we drop any duplicates that most likely reflect piggyback loans in order to avoid double-counting names. The term `piggyback' refers to a second mortgage that is made at the same time as the main mortgage in order to reduce the down payment without paying for private mortgage insurance. We identify as piggyback loans about one percent of the observations across the datasets.
- Drop all applications where information on race and ethnicity was not provided (e.g., Internet applications). Similarly, drop all applications with missing values for race or ethnicity.
- Keep only applications with either one applicant or two applicants that have the same race and ethnicity. The reason for keeping only joint applications with the same race/ethnicity is because in a few cases we observe inversion in the data entry for the applicants' gender, which may well extend to the race and ethnicity.
- Drop any applicant names that correspond to companies and trusts. These are rare cases, since they would typically have missing information on race and ethnicity. Such cases are easily detectable since the applicant name includes strings such as ‘Inc.,’ ‘LLC,’ and ‘Trust.’
- Separate the first and last names, and drop the middle name, which is typically an initial. Also, drop any suffixes (e.g., ‘III,’ ‘IV,’ ‘Sr.,’ and ‘Jr’) and convert all strings to uppercase.
- Drop any applications where either the surname or the first name contains a numeric character.
- Drop any observations where either the surname or the first name contains only one alphabetic character.
- Any intervening blanks and hyphens are deleted. This step is also used by the Census in creating aggregate information on surnames^3^. For instance, VAN HALEN becomes VANHALEN and GARCIA-MARTINEZ becomes GARCIAMARTINEZ.

### Finalizing the first name list

Based on the aforementioned steps, the three datasets yield a total of 2,663,364 first name observations and 1,925,186 surname observations. The latter is smaller because of the joint applications which typically contain the same surname and two different first names. Also, these numbers are generally consistent with the fact that about half of the mortgage applications in the United States have a single applicant. Furthermore, the 2,663,364 first name observations reflect 91,526 distinct first names. We combine the three datasets into a single dataset, and finalize the unique list of first names based on the following steps:

- For each first name and surname (i.e., separately), we calculate the proportion for each of the six categories.
- We do not consider names whose proportions are based on fewer than 30 observations. The only exception is when the proportion is unity for a single category and zero for all other five categories, and it is based on 15–29 observations. In this way, we are able to capture strictly ethnic names. For instance, in our list the first name TOMISLAV is based on 16 observations that are all assigned to the ‘NH White’ category. Similarly, TAKASHI is based on 17 observations that are all assigned to the ‘NH Asian or Native Hawaiian or Other Pacific Islander’ category. From a statistical perspective, when the sample contains 15 observations then the lower bound for the exact binomial 90 percent confidence interval for the probability of that single category is 0.82, indicating that the expected population probability remains high.

The final outcome is two lists with 4,250 first names and 11,299 surnames and their corresponding proportions for race and ethnicity. For the great majority of cases, these proportions are calculated using more than 30 observations (see Table 2). In fact, for about two-thirds of the first names we have 50 or more observations. From a coverage perspective, the two lists cover the vast majority of the popular names. Specifically, using the 1990 Census list with the most popular first names for males and females, we find that our list of first names captures 97.0 percent of the top-1,000 female first names and 95.8 percent of the top-1,000 male first names. The level of data coverage is remarkable since the list of 4,250 first names would reflect 85.6 percent of the U.S. population, based on the 1990 Census information on first name frequencies. The high level of coverage from our first name list is also demonstrated by the fact that although it corresponds to a small fraction of the initial first-name list (4,250 out of 91,526 first names), it covers 92 percent of the first name observations (2.45 million out of 2.66 million observations). Concerning our list of surnames, which is used only for validation purposes (see ‘Technical Validation’ section), it contains 80.4 percent of the top 10,000 surnames and reflects 67.5 percent of the U.S. population, according to the 2010 Census information.

Overall, the combination of the reasonable sample sizes used to calculate the proportions and the wide population coverage offer an initial indication that the first name list could be helpful in correctly identifying individuals’ race or ethnicity.

### Additional information on the data merging methodology

This section offers details on the methodology used to merge HMDA with the Property Transaction data offered by DataQuick, a provider of real estate information. In particular, we performed the following four steps:

- *Choose the baseline HMDA data*. We use the originated loans included in the HMDA dataset for 2010.
- *Organize the DataQuick data on property transactions*. We consider only records that involve either home purchase or refinance. Similarly, we drop any multiple parcel transactions. Furthermore, we consider only arms-length transactions, thus excluding any transfer of property from parents to children or into trusts. Although these transfers would not be reflected in HMDA, they may obstruct the matching process by creating false matches. In a related fashion, we drop cases of quitclaim deeds, which are rarely used for traditional property sales, but rather for transferring properties to family members, moving properties into trusts, or for divorce proceedings. We also exclude any quasi-arm-length home purchases (e.g., a property transfer between siblings). To clarify loan types, we assume that any records in DataQuick that are not explicitly coded as FHA or VA loans are conventional loans. From a data integrity perspective, we ensure that the filing date for the transaction falls within the time period for which DataQuick claims that it has municipality recorder information for the relevant municipality. Finally, we drop a small number of transaction records with missing Federal Information Processing Standards (FIPS) information in DataQuick.
- *Merge HMDA with DataQuick*. The matching criteria are identical loan amount (rounded to the nearest thousand), property type, loan type, loan purpose, and FIPS code (census tract), as well as the absolute difference between the HMDA action date and the DataQuick transfer date to be less than 30 days. Merging for all these criteria occurs in a single step. Notably, both HMDA and DataQuick use the 2000 census tract definitions, and as a result the property location can be matched at the census tract level. Observations with missing values for the matching parameters were not considered during the matching exercise. We ensure accurate matching by keeping only one-to-one matches between HMDA and DataQuick.
- *Finalizing the merged dataset and ensuring data quality*. Following the aforementioned steps, we obtain a merge rate of 26 percent. From the resulting dataset, we drop a relatively small number of observations with missing values for the borrower's (borrowers') name. These deleted observations account for less than 1 percent of the dataset. We also drop any observations from the particular lender for whom we have the enhanced HMDA dataset for 2010. In fact, we check whether these matched observations from HMDA-DataQuick actually belong to the particular lender and involve the said individuals. We find that 94 percent do so, while most of the remaining observations involve partially erroneous name entries either in the lender dataset or in the property transaction records (e.g., JOHN MILLER and JOHN MILER). This test reaffirms the quality of data used to create the first name list.

Finally, we take one additional measure to ensure merging accuracy by checking *ex-post* whether the lender identifier in HMDA (i.e., respondent_id variable) is the same with the lender identifier in DataQuick (i.e., sr_lndr_first_name_1 variable). We confirm this identity for 90.2 percent of the merged dataset, illustrating the quality of the matching process. Even though the remaining 9.8 percent of the matches could still be valid (e.g., the occurrence of mergers or acquisitions could explain the lender name change), we opt to drop them from the final merged dataset.

### Availability of data sources and code

Unfortunately, we cannot provide access to the data sources due to their proprietary nature. All procedures implemented in this project were written in Stata (version 13.1) do-files. These do-files cannot be shared because their content (e.g., variable names) might reveal the identity of the lenders. Nevertheless, the code is straightforward and its various steps were described in detail in this section, thus making it possible for a third party to exactly repeat our methodology.

## Data Records

The data with race/ethnicity information for 4,250 first names can be downloaded at *Harvard Dataverse* (Data Citation 1). We confirm that we have appropriate approval to share this data. The data can be downloaded as an Excel file (firstnames.xlsx), which contains eight fields as shown in Table 3. The choice of summarizing the first names' demographic information into six mutually exclusive racial and Hispanic origin groups was made to retain consistency with the 2010 and 2000 Census Bureau's list of surnames. Finally, unlike the 2010 (2000) Census' surname list which is based on 266 (242) million individuals, we do not suppress a field that corresponds to fewer than five counts, since our data source is only a small fraction (about 1 percent) of the U.S. adult population.

## Technical Validation

### Comparison of surname information

We will use our observed race/ethnicity proportions for surnames as an indirect way to test the quality of the process. More specifically, we compare our classification for surnames against the respective 2010 Census classification for surnames. The intuition is that if these two classifications are very similar, then the classification for first names should be reliable too.

For the purposes of this test, we randomly choose 5,000 surnames from our list and we compare their observed probabilities with the respective population probabilities from the 2010 Census' list of surnames^5^. For comparability purposes, we consolidate the ‘NH American Indian or Alaska Native’ and ‘NH Multi-race’ categories into a single category (‘NH Other’) not only because they reflect a very small portion of the mortgage population (about 1 percent) and the total U.S. population (about 3 percent), but also because we want to ensure that the results are not affected by empty cells. We then perform a Fisher's exact test for each surname's 2x5 contingency table between the two lists and the five categories (i.e., Hispanic, NH White, NH Black, NH Asian or Pacific Islander, NH Other). The null hypothesis is that a surname's classification is not different across the two lists. The results indicate a high level of similarity between the Census surname classifications and our own surname classifications in terms of race and ethnicity. More specifically, we find that the null hypothesis is not rejected at the 1 percent (5 percent) level of statistical significance for 80.4 percent (75.2 percent) of the surnames in the sample of 5,000 surnames.

### Descriptive statistics from a validation dataset

For the purposes of this validation exercise, we use a lender's mortgage application data from 2012. The reason for choosing an actual loan application dataset is to be able to offer a realistic environment for testing the first name race/ethnicity information in the context of fair lending examinations. For consistency, we follow the same steps as those outlined in the ‘Methods’ section to reduce the lender's dataset into just names and race/ethnicity categories. For practical purposes, we unify the ‘NH American Indian or Alaska Native’ and ‘NH Multi-race’ categories into one (‘NH Other’) since together they represent only about 1 percent of the sample. To protect the identity of the lender, we draw a random sample of 20,000 applicants. The final dataset has 20,000 observations and contains four variables indicating each applicant's first name, surname, race and ethnicity. Table 4 presents the applicants in the dataset by race and ethnicity.

We upload the mortgage application dataset and merge the population-based proportions for surnames from the Census’ 2010 list and the sample-based proportions from our list of first names. Table 5 shows that the merge rate for the Census' surnames was 85.6 percent, which is consistent with recent studies that test race/ethnicity proxies in the context of loan applications^12^. Notably, a similar merge rate was obtained for our first name demographic information (86 percent). Of particular note is the small overlap between the missing values for first name information and surname information. Based on the aforementioned, first name demographic information not only has equally wide coverage as the surname demographic information, but it can also help with imputing missing values for surname demographic information.

**Figure 1:**
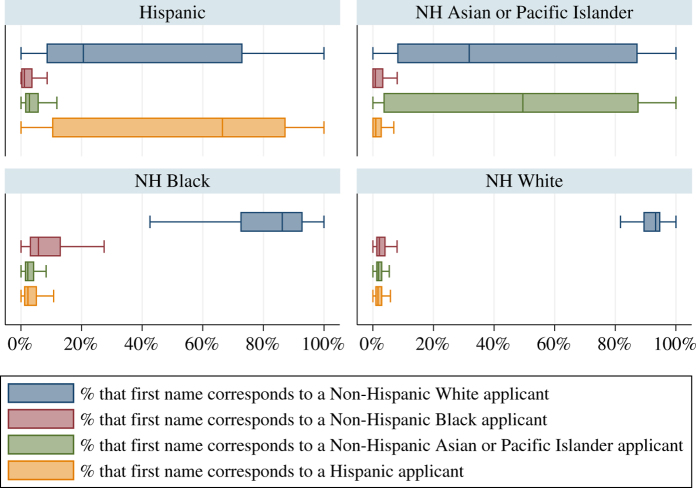
First Names -- Distribution of proportions across race/ethnicity categories. These four categories (i.e., Hispanic; NH White; NH Black; NH Asian, Native Hawaiian or Other Pacific Islander) reflect 99 percent of the validation dataset. The empirical distributions are presented using horizontal box plots, with the *x*-axis denoting proportions. The line inside the box denotes the median, while the interior of the box denotes the interquartile range (IQR). In each box, the upper adjacent value is equal to the upper quartile plus 1.5*IQR, while the lower adjacent value is equal to the lower quartile minus 1.5*IQR. For presentation purposes, we exclude outliers.

Figure 1 offers insights into the empirical distribution of the proportions for the first names vis-à-vis the actual race/ethnicity category of the applicants. For instance, for applicants whose reported race/ethnicity is ‘NH White’ we observe that the ‘NH White First Name’ proportions are highly leptokurtic and concentrated above the 85 percent mark. In contrast, for the same applicants the empirical distribution of the ‘NH Black First Name’ proportion or the ‘Hispanic First Name’ proportion is again highly leptokurtic, but concentrated around the 5 percent mark. These attributes make our first name demographic information a robust predictor of NH White applicants. First name demographic information could also be helpful, albeit to a lesser extent, for Hispanic applicants and NH Asian or Pacific Islander applicants, where the respective first name proportions are relatively indicative. For example, half of the Hispanics in our sample have a first name that has a proportion of 65 percent or higher for belonging to a Hispanic individual. However, first names do not seem to distinguish NH Black applicants well, since most of the empirical distribution of the ‘NH Black First Name’ proportion for those applicants lies below the 20 percent mark.

**Figure 2:**
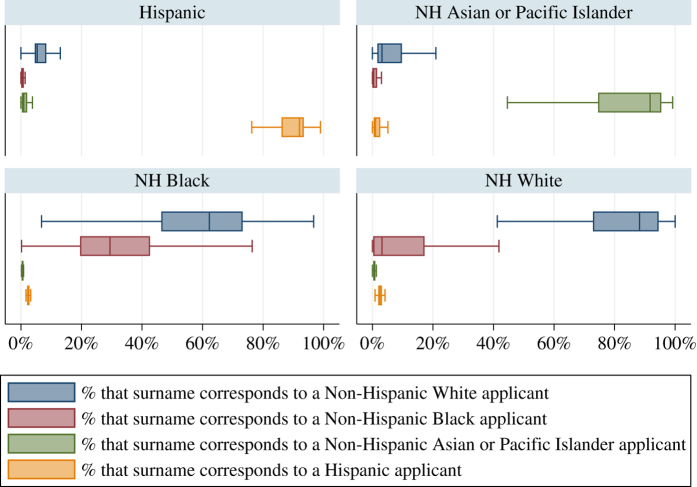
Surnames -- Distribution of proportions across race/ethnicity categories. These four categories (i.e., Hispanic; NH White; NH Black; NH Asian, Native Hawaiian or Other Pacific Islander) reflect 99 percent of the validation dataset. The empirical distributions are presented using horizontal box plots, with the *x*-axis denoting proportions. The line inside the box denotes the median, while the interior of the box denotes the interquartile range (IQR). In each box, the upper adjacent value is equal to the upper quartile plus 1.5*IQR, while the lower adjacent value is equal to the lower quartile minus 1.5*IQR. For presentation purposes, we exclude outliers.

For comparison purposes, Figure 2 offers insights into the empirical distribution of the population-based proportions for the surnames vis-à-vis the actual race/ethnicity category of the applicants. It is evident that the surname information offers different strengths compared with the first name information. In particular, for applicants whose reported ethnicity is ‘Hispanic’ we observe that the ‘Hispanic’ surname population-based proportions are leptokurtic and concentrated above the 80 percent mark. In a modeling framework, the relative strengths of each set of information could interplay in order to improve the accuracy of the race/ethnicity assignment.

### Assessing alternative data sources

We opted to use mortgage application data because the quality of the race/ethnicity information and the wide geographic coverage of that data could offer a reasonable approximation of the demographic information related to first names in the U.S. population. The choice not to use state-level data on vital statistics, namely birth or death records, and voter registration was based on a number of factors. Concerning vital statistics, many states limit the scope of their data sharing only to health authorities. Also, the definition of race/ethnicity categories and the availability of race/ethnicity information may vary across states and across time. Similarly, rather than offering the raw data, many states offer the data only in some aggregated form, excluding any names with few observations. Moreover, data from birth records do not reflect information for foreign-born population, which currently constitutes about 13 percent of the total U.S. population, while any inferences from death records may not be relevant to the current population. In terms of voter registration data, the vast majority of states either do not have information on the race and ethnicity of the voters, or have restrictions on the use of the data, or both. Moreover, in terms of data integrity when combining data across states, there may be concerns as data maintenance and voter purge practices vary substantially across election boards. For instance, North Carolina and Florida are two populous states where voter registration data are both available with minimal restrictions and contain race/ethnicity information. However, even for these two states, the voter registration data may not be representative of both the state and the U.S. population. For example, the portion of Hispanics reported in the 2017 Voter Registration Statistics for North Carolina was about 2.5 percent, when Hispanics constitute about 7 percent of the state's adult population. The same holds for Florida, as the portion of Hispanics in the state’s voter registration data and the state's adult population is about 16 percent and 24 percent, respectively. Finally, if we use the information on active voters in the North Carolina voter registration data or the Florida voter registration data (or the combined data) and repeat the surname comparison test presented in the 'Technical Validation' section, we find the respective level of surname classification accuracy vis-à-vis the 2010 Census to be lower than the one reported with our sample.

## Usage Notes

In a number of important policy areas, such as drug effectiveness and discrimination in financial and health services, there has been a growing need to accurately identify the race and ethnicity of individuals when there is no requirement for collecting such information. As a result, practitioners and researchers are drawing inferences from relevant information that is already present in various documents (e.g., medical prescriptions, loan applications). This process requires publicly available data that offer insights on how the relevant information, such as a person's surname and address, is associated with a specific race or ethnicity.

The assignment accuracy depends on two factors: the availability of inputs from which one can infer race or ethnicity, and the methodology of combining these inputs into a single probability. This paper operates in the first direction, by offering race/ethnicity information for first names, thus addressing a part of the full name that had been previously underutilized, if not neglected. As such, this paper offers the opportunity for various methodologies to incorporate one more parameter, *first name*, in order to increase their race/ethnicity classification accuracy. To the best of our knowledge, this is the first such effort, as the Census has provided race/ethnicity information for surnames only. The use of first names can increase the classification accuracy either *directly*, by adding first names in the model, or *indirectly*, by improving the imputation of missing surname information. Although first names may offer a noisier signal than surnames as they are influenced by family preferences for cultural assimilation and cycles in name popularity^13^, the first name is perhaps the last important associated information that is readily available, since any remaining readily available information (e.g., date of birth, phone number) is generally uncorrelated with race or ethnicity.

Our list of first names is not based on Census data for the entire U.S. population, but on mortgage applications. As a result, its demographic information is more representative when the sample at hand has characteristics that are closer to those of mortgage applicants (e.g., adult population, employed population). Perhaps the most effective use of our first name list would be in the context of fair lending, both for non-mortgage and mortgage products. Non-mortgage consumer finance (e.g., auto loans, credit cards) is a natural area of application, given that it does not fall under the HMDA reporting statute; hence, lenders are not collecting information on the applicants' race and ethnicity. Also, in terms of mortgage products, the first name list could be used to evaluate the missing values in the race and ethnicity fields for a specific lender's LAR. Given the increasing popularity of online or telephone mortgage applications, it is important to evaluate whether race and ethnicity information is missing in a non-random fashion that could introduce a sample selection bias. Overall, this paper suggests the use of first names as an additional piece of associated information to infer race and ethnicity, and provides a list with 4,250 first names and their respective proportions across six race/ethnicity categories, which are also used in the 2010 and 2000 Census surname lists.

## Additional information

**How to cite this article**: Tzioumis, K. Demographic aspects of first names. *Sci. Data* 5:180025 doi: 10.1038/sdata.2018.25 (2018).

**Publisher**’**s note**: Springer Nature remains neutral with regard to jurisdictional claims in published maps and institutional affiliations.

## Supplementary Material



## Figures and Tables

**Table 1:** Classifying applicants into OMB categories based on race and ethnicity information in HMDA.

**OMB Categories**	**HMDA ethnicity variable**	**HMDA race variable**
Hispanic or Latino	Hispanic or Latino	White
Hispanic or Latino	Hispanic or Latino	Black or African American
Hispanic or Latino	Hispanic or Latino	Asian
Hispanic or Latino	Hispanic or Latino	Native Hawaiian or Other Pacific Islander
Hispanic or Latino	Hispanic or Latino	American Indian or Alaska Native
Hispanic or Latino	Hispanic or Latino	Non-missing secondary race variable
White	Not Hispanic or Latino	White
Black	Not Hispanic or Latino	Black or African American
Asian/Nat. Haw./Other Pac. Isl.	Not Hispanic or Latino	Asian
Asian/Nat. Haw./Other Pac. Isl.	Not Hispanic or Latino	Native Hawaiian or Other Pacific Islander
American Indian/Alaska Native	Not Hispanic or Latino	American Indian or Alaska Native
Multi-race	Not Hispanic or Latino	Non-missing secondary race variable

**Table 2:** Sample size, by name.

**Samplesize range**	**First names**		**Surnames**	
	**Frequency**	**Percentage**	**Frequency**	**Percentage**
[15–29]	376	8.8%	3,522	31.2%
[30–49]	1,159	27.3%	3,060	27.1%
[50–99]	1,006	23.7%	2,373	21.0%
[100–249]	739	17.4%	1,453	12.8%
[250–499]	337	7.9%	511	4.5%
[500–999]	225	5.3%	225	2.0%
1000+	408	9.6%	155	1.4%
*Total*	4,250	100.0%	11,299	100.0%
The table presents the number of observations used in calculating the proportions for race/ethnicity for the 4,250 first names and 11,299 surnames in our list. The minimum number of observations for each name is 30, except for cases in which the proportion is unity for a single category and it is based on 15-29 observations. The list of 4,250 first names is based on 2,449,240 first name observations, while the list of 11,299 surnames is based on 1,240,098 surname observations.				

**Table 3:** Description of fields.

**Field**	**Description**
firstname	First name
obs	Number of occurrences in the combined mortgage datasets
pcthispanic	Percent Hispanic or Latino
pctwhite	Percent Non-Hispanic White
pctblack	Percent Non-Hispanic Black or African American
pctapi	Percent Non-Hispanic Asian or Native Hawaiian or Other Pacific Islander
pctaian	Percent Non-Hispanic American Indian or Alaska Native
pct2prace	Percent Non-Hispanic Two or More Races

**Table 4:** Composition of validation dataset in terms of race and ethnicity.

**Category**	**Asian or Pac. Isl.**	**Black**	**White**	**Other**	***Total (Row)***
Non-Hispanic	10.9%	10.5%	66.8%	0.8%	89.0%
Hispanic	0.3%	0.3%	10.0%	0.4%	11.0%
* Total (Column)*	11.2%	10.8%	76.8%	1.2%	100.0%

**Table 5:** Coverage for first name and surname demographic information.

	First Name Information		*Total (Row)*
	Present	Missing	
Present Surname Information	15,159	2,131	17,290
Missing Surname Information	2,036	674	2,710
* Total (Column)*	17,195	2,805	20,000
